# DRP5 is involved in cancer cell growth and predicts poor prognosis in human osteosarcoma

**DOI:** 10.1002/cam4.1009

**Published:** 2017-04-04

**Authors:** Lin Wang, Weihai Liu, Hengtao Tang, Xianbiao Xie, Changye Zou, Yongqian Wang, Zhenhua Gao, Junqiang Yin

**Affiliations:** ^1^ Department of OncologyGuangzhou Red Cross HospitalMedical CollegeJinan UniversityGuangzhouChina; ^2^ Department of Musculoskeletal OncologyThe First Affiliated Hospital of Sun Yat‐Sen UniversityGuangzhouChina; ^3^ Department of OrthopedicsThe First Affiliated Hospital of Zhengzhou UniversityZhengzhouChina; ^4^ Department of RadiologyThe First Affiliated Hospital of Sun Yat‐Sen UniversityGuangzhouChina

**Keywords:** Animal model, DRP5, invasion, lung metastasis, migration, osteosarcoma, overall survival

## Abstract

Osteosarcoma is an extremely aggressive primary malignant bone tumor of childhood. Collapsin response mediator proteins (CRMPs), which are highly expressed in the developing nervous system, were recently shown to be associated with cancer development. However, the relationship between DRP5 (CRMP5) and osteosarcoma has not been evaluated. In this study, we investigated the role of DRP5 in the regulation of osteosarcoma growth. DRP5 mRNA and protein levels were significantly upregulated in human osteosarcoma cell lines and associated with increased migration and invasion. Genetic knockdown of DRP5 markedly suppressed the expression of matrix metalloproteinase (MMP)‐2 and MMP‐9. DRP5 silencing significantly inhibited osteosarcoma cell growth in vitro and in a xenograft mouse model in vivo. Microarray immunohistochemical analysis of osteosarcoma specimens and Kaplan–Meier analysis showed that patients with high DRP5 protein expression had shorter overall survival than those with low DRP5 levels. Taken together, these results suggest that DRP5 plays a critical role in the regulation of osteosarcoma and could be a potential therapeutic target and prognostic factor in osteosarcoma.

## Introduction

Osteosarcoma is the most common primary bone malignancy in children and adolescents, accounting for approximately 2.4% of all malignancies in pediatric patients 1. Because of its high propensity for distant metastasis, osteosarcoma is one of the leading causes of cancer‐related death in adolescents. Metastasis, a major cause of treatment failure in cancer, involves many complex processes such as cell migration, angiogenesis, adhesion, and proliferation 2. Although osteosarcoma patients may initially respond to chemotherapy, those with metastatic or recurrent disease have extremely poor survival rates 3, 4, 5. It is therefore critical to identify the mechanisms underlying osteosarcoma development and progression.

Collapsin response mediator proteins (CRMPs) comprise five isoforms (CRMP 1–5), all of which are highly expressed in developing neurons 6, 7, 8. DRP5 (CRMP5), which was first identified as a CRMP‐associated protein and designated as CRAM, shares the lowest homology with other CRMPs 9. Accordingly, DRP5 regulates the dynamics of filopodia, growth cone development, dendritic development, and synaptic plasticity 10, 11 by interacting with other proteins such as Fes/Fps tyrosine kinase 12 and the mitochondrial protein septin 13. Because of their involvement in regulating cell migration by interacting with the cytoskeleton 14, 15, 16, CRMP family proteins have generated interest in recent years for their potential role in cancer. The expression of the long form of CRMP‐1, LCRMP‐1, in patients with nonsmall‐cell lung cancer (NSCLC) 17 and gastric cancer 18 was associated with poor clinical outcomes 19. CRMP‐2 was identified as a prognostic marker and candidate therapeutic target in NSCLC 20. Reduced CRMP‐2 expression and elevated expression of nuclear phosphorylated CRMP‐2 were associated with breast cancer progression 21. A neuronal autoantibody specific for DRP5 was identified and suggested as a marker of lung cancer and thymoma‐related autoimmunity 22. However, the role of DRP5 in osteosarcoma remains largely unknown. The high propensity of osteosarcoma to metastasize to the lung and the involvement of DRP5 in lung cancer underscore the need to investigate the potential role of DRP5 in the development of osteosarcoma. Here, we examined the expression of DRP5 in samples from osteosarcoma patients and cultured cells in vitro and in a mouse model in vivo.

## Methods and Materials

### Ethics statement

All human and animal experiments were approved by the Medical Ethical Committee for Clinical Research and Animal Trials of the First Affiliated Hospital of Sun Yat‐Sen University. All experiments using human specimens were performed in accordance with the Declaration of Helsinki. Tissue samples were obtained from osteosarcoma patients in the First Affiliated Hospital of Sun Yat‐Sen University. Informed consent was obtained from all participants prior to the study. All animal procedures were performed following the humane care guidelines of the Chinese National Institute of Health, and the protocols were approved by the committee of Sun Yat‐Sen University (Approval Number: 2016[148]).

### Cell culture and shRNA stable cell line

Human hFOB1.19 osteoblasts and the osteosarcoma cell lines SAOS2 and MG63 were purchased from Cell Bank of Shanghai Institute of Cell Biology, Chinese Academy of Sciences (Shanghai, China). Cells were cultured in Dulbecco's minimal essential medium (DMEM) supplemented with 10% fetal bovine serum (FBS), streptomycin (100 *μ*g/mL), and penicillin (100 U/mL) in a humidified atmosphere with 5% CO_2_ at 37°C. The pLKO.1 puro vector (Addgene, Cambridge, MA) with a U6 promoter was used for the construction of recombinant lentiviral plasmid encoding sh‐DRP5. The shRNA sequence was as follows: 5′‐GTGGACGCTTATGAGAAGT‐3′. HEK 293T cells were cotransfected with pLKO.1 puro and packaging vectors. The supernatant was harvested at 48 and 72 h after transfection. MG63 cells were infected with shDRP5 and shCtrl (negative control, NC) in the presence of 5–10 *μ*g/mL polybrene; after 48 h, cells were incubated in medium with 2 *μ*g/mL puromycin for 12 days to generate osteosarcoma‐MG63 shDRP5 stable cell lines.

### In vivo tumor model

Animal experiments were performed under approval from the Committee on Animal Research of Sun Yat‐Sen University, Guangzhou. To investigate the effect of DRP5 inhibition on osteosarcoma cell growth in vivo, cells (2 × 10^6^) from the osteosarcoma MG63‐shDRP5 and MG63‐shCtrl stable lines were inoculated subcutaneously into the flanks of nude mice. Six mice (one mouse in the shDRP5 group died) were included in each group; tumors were measured during the study period, and tumor volume was calculated using the formula for a prolate spheroid (V = 4/3 *π*a^2^b) 23. Four weeks later, mice were sacrificed, and tumor weight was recorded.

### Migration and invasion assay

Cell migration assays were performed using a Transwell system (Millipore, MA). Cells were cultured in serum‐free DMEM for 24 h, trypsinized, and resuspended in serum‐free DMEM. Cells were seeded in the upper chamber of the Transwell insert and incubated with 0.5% DMSO or deguelin (10 and 15 *μ*mol/L), and 90% DMEM containing 10% FBS was added to the lower chamber and incubated for 48 h. The remaining cells in the upper chamber were removed. Cells migrated into the lower surface of the filter were fixed and stained with 2% crystal violet for quantification. For the invasion assay, the Transwell filter membrane was coated with Matrigel (Becton Dickinson Bioscience, MA). Cell numbers were expressed as the mean ± SD. At least three experiments were performed and each was performed in triplicate.

### MTS assay

In vitro* *cell proliferation was assessed with the CellTiter 96^®^ AQueous One Solution Cell Proliferation Assay Kit (Promega, Madison, WI) according to the manufacturer's instructions. In brief, in each group, cells were allowed to grow for 6, 24, 48, and 72 h. At harvesting, 20 *μ*L of CellTiter 96 AQueous One Solution reagent was added to each well in a total volume of 100 *μ*L of medium and incubated for 3–4 h. Absorbance was measured at 490 nm using an ELISA plate reader. The growth rate was calculated from the absorbance, and the readings at 6 h time points in each group were set to 100%.

### Cell cycle assay by flow cytometry

Cells plated in six‐well plates were washed twice with PBS and fixed in 70% ethanol at 4°C overnight. Then, cells were incubated with propidium iodide at room temperature for 1 h and analyzed by flow cytometry using a FACScan flow cytometer (BD Biosciences, Mountain View, CA).

### Wound healing assay

MG63 cells were grown in six‐well dishes until confluence. The confluent monolayer was scraped with a pipette tip. Then, the cells were washed twice with PBS and cultured in a 5% CO_2_ incubator for 48 h at 37°C. Images of each well were acquired immediately and at 24 and 48 h in four random fields using an inverted fluorescence microscope (Nikon Corporation, Tokyo, Japan) at ×100 magnification. Wound closure was expressed as the average ± SEM of the difference between the measurements at time zero and the 24–48 h time period.

### Human osteosarcoma specimens

Studies of osteosarcoma patients were approved by the Institutional Review Board of Sun Yat‐Sen University. Informed consent was signed before sample collection. In this study, osteosarcoma samples were obtained from 108 patients between January 2005 and December 2009, including 21 biopsy samples and 87 samples from surgical resections. Biopsy samples were fixed for 12 h with 10% formaldehyde when obtained and then paraffin‐embedded. Surgical resections were fixed with 10% formaldehyde within 3 h after resection. All samples were subjected to hematoxylin and eosin staining to confirm the diagnosis of osteosarcoma.

### Immunohistochemistry

The specific anti‐DRP5 antibody for western blotting and immunohistochemistry was purchased from Abcam (Cambridge, MA). For immunohistochemistry, paraffin sections were treated with hydrogen peroxide to inactivate endogenous peroxidases. Antigen retrieval was performed in a microwave in 10 mmol/L citrate buffer at pH 6.0. Sections were fixed with paraformaldehyde followed by permeabilization and blocking. Sections were then incubated in anti‐DRP5 antibody overnight at 4°C, and a secondary antibody was used to detect protein expression. Immunostaining was analyzed with the Super Sensitive Non‐Biotin Polymer HRP Detection System according to the manufacturer's instructions (BioGenex, San Ramon, Canada).

### Western blotting

Cells were lysed in sample buffer and subjected to sodium dodecyl sulfate‐polyacrylamide gel electrophoresis as described previously 24. Primary antibodies against DRP5, matrix metalloproteinase (MMP)‐2, and MMP‐9 were obtained from Abcam. GAPDH (Abcam) was used as the loading control. After washing with PBS containing 0.1% Tween‐20 five times, the membrane was incubated with the appropriate horseradish peroxidase‐conjugated secondary antibodies (Amersham Biosciences, Uppsala, Sweden) for 1 h, and bands were detected by enhanced chemiluminescence (Amersham). In all cases, the background intensities were first subtracted. Densitometric values were normalized to GAPDH levels.

### Total RNA extraction and RT‐PCR

Total RNA was isolated using the Trizol reagent (Invitrogen, Carlsbad, CA) according to the manufacturer's protocol, and the mRNA levels of DRP5 were evaluated by RT‐PCR. First, RNA was reverse transcribed using RT‐PCR Quick Master Mix (Toyobo Biochemicals, Osaka, Japan) according to the manufacturer's protocol. The results were normalized to the expression of GAPDH. The oligonucleotide primers used were as follows: DRP5‐forward, 5′‐TTGTGGACGCTTATGAGAAGTG‐3′; DRP5‐reverse, 5′‐CTCACCAGTGTCTCCATTTCTG‐3′; GAPDH‐forward, 5′‐ GAGTCAACGGATTTGGTCGT‐3′; GAPDH‐reverse, 5′‐ GACAAGCTTCCCGTTCTCAG‐3′.

### Statistical analysis

All experiments were repeated at least three times, and the results are presented as the mean ± SD. Analyses of significance were performed using Student's *t*‐tests or one‐way ANOVAs, followed by Bonferroni corrections. ROC curve analysis was used to define the cutoff score for high expression of DRP5. Survival curves were built using the Kaplan–Meier method and analyzed using the log‐rank test. SPSS 20.0 software was used for statistical analyses. A value of *P *<* *0.05 is considered statistically significant.

## Results

### DRP5 is upregulated in human osteosarcoma cell lines

**Figure 1:**
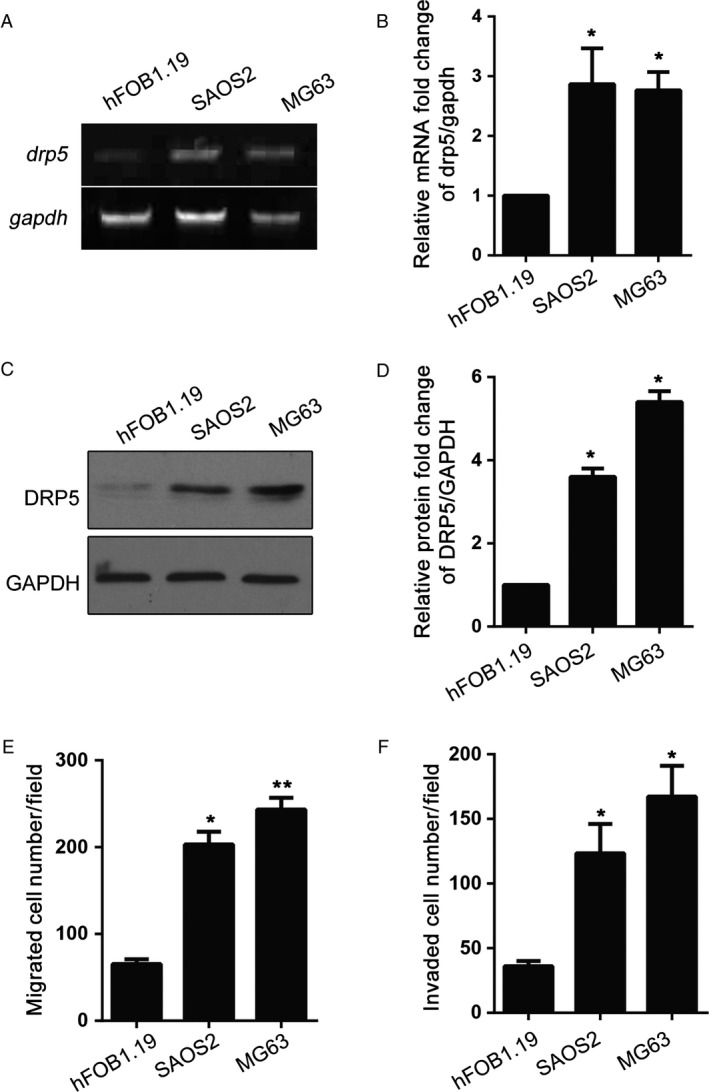
Increased DRP5 expression is related to invasion and migration in osteosarcoma cell lines DRP5 was upregulated in osteosarcoma cells at both mRNA (**A, B**) and protein levels (**C, D**). mRNA and protein levels were measured by RT‐PCR and western blotting, respectively. The migration (**E**) and invasion (**F**) abilities of osteosarcoma cell lines were determined. All experiments were performed at least three times, and data are expressed as the mean ± SD. * denotes P < 0.05, ** denotes P < 0.01, versus hFOB1.19.

To evaluate the role of DRP5 in osteosarcoma, the mRNA and protein expression levels of DRP5 were measured by western blotting and RT‐PCR in hFOB1.19 osteoblasts and the osteosarcoma cell lines SAOS2 and MG63. DRP5 mRNA levels were higher in osteosarcoma cell lines than in the osteoblast hFOB1.19 line (Fig. 1A). DRP5 mRNA levels normalized to GAPDH expression are shown in Figure 1B. Assessment of protein levels showed a similar pattern to that of mRNA levels (Fig. 1C and D). These results indicated that DRP5 is significantly overexpressed in osteosarcoma, suggesting the involvement of DRP5 in osteosarcoma development. To better understand the potential function of DRP5 in osteosarcoma cells, migration and invasion assays were performed in hFOB1.19, SAOS2, and MG63 cells. As shown in Figure 1E and F, the number of migrated and invaded cells was greater in osteosarcoma cell lines than in the normal osteoblast cell line. This increased migration and invasion capacity was consistent with the upregulation of DRP5 in osteosarcoma cell lines.

### DRP5 silencing suppressed migration and invasion of osteosarcoma cells

**Figure 2:**
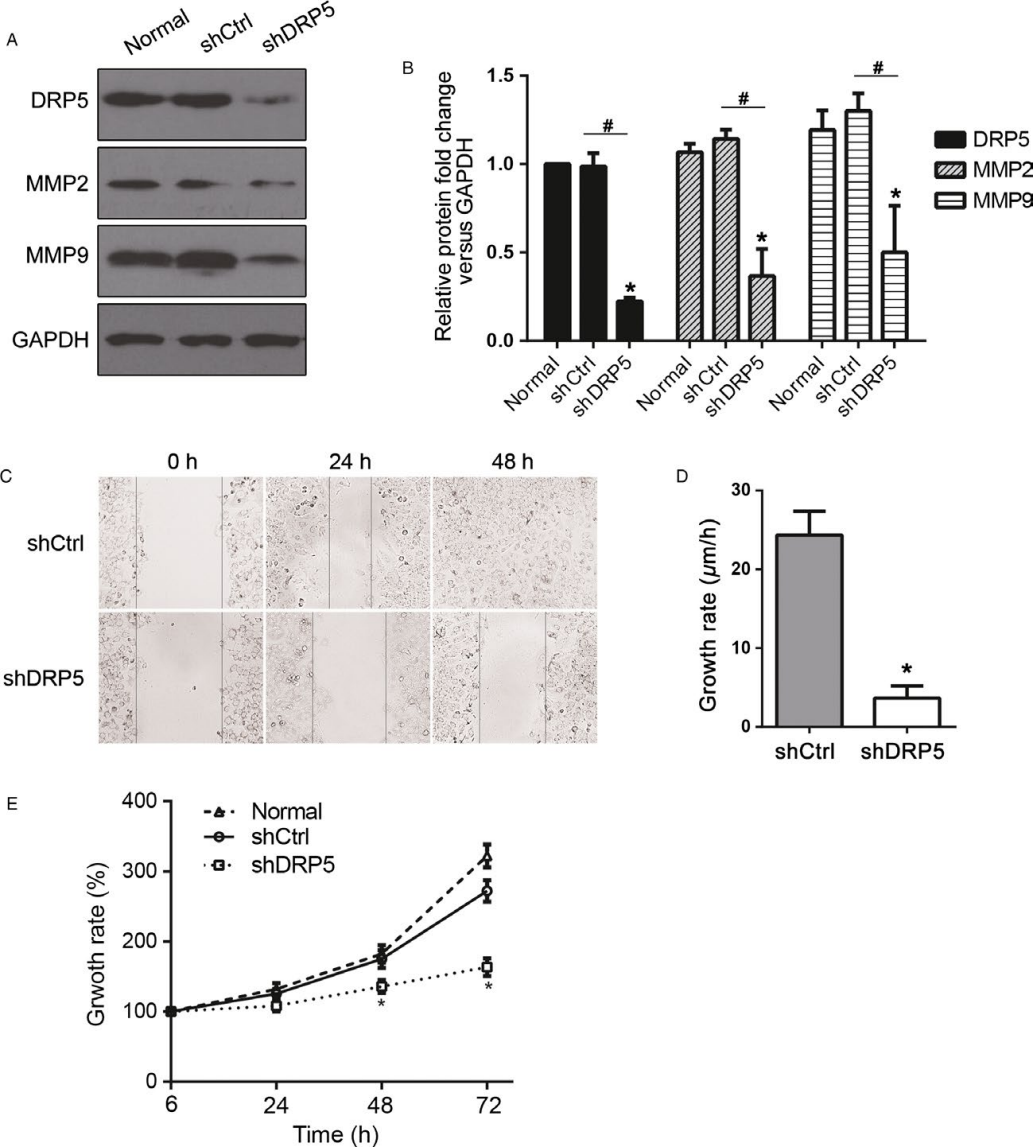
Reduced cell growth in DRP5 knockdown cells. (A, B) The protein levels of DRP5 and the invasive factors MMP‐2 and MMP‐9 were detected in shRNA stable cell lines. Data normalized to GAPDH are shown (**B**). (**C**) Representative wound healing assays and (**D**) analysis of growth rates are shown. All experiments were performed in triplicate, and results are expressed as the mean ± SD. * denotes P < 0.05 versus normal cells. # denotes P < 0.05 versus shCtrl cells.

**Figure 3:**
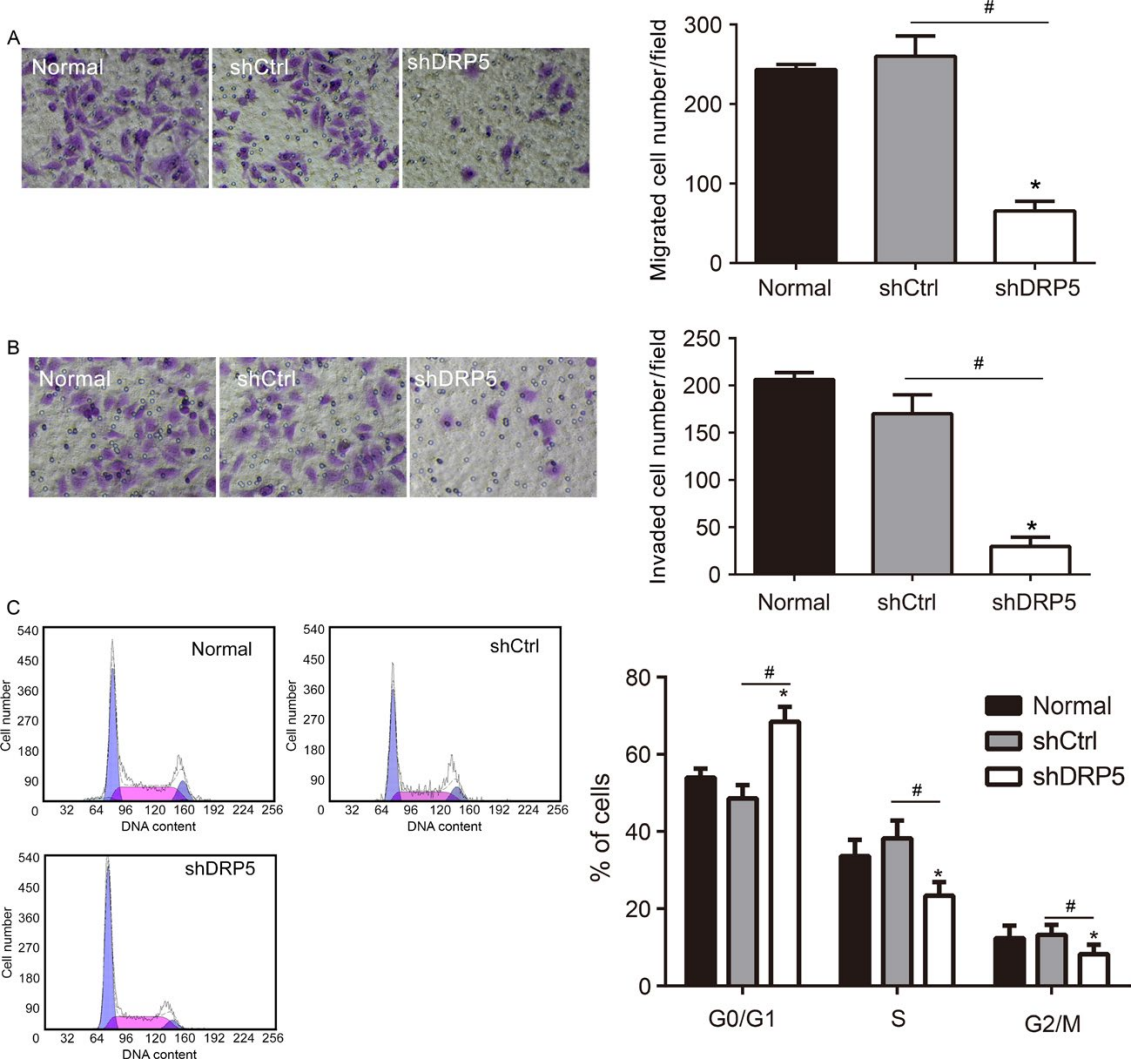
Reduced cell migration and invasion in DRP5 knockdown cells. Normal, shCtrl, and shDRP5 MG63 cells were subjected to migration (A), invasion (B), and flow cytometry (C) assays. All experiments were performed in triplicate, and results are expressed as the mean ± SD. * denotes P < 0.05 versus normal cells. # denotes P < 0.05 versus shCtrl cells.

Because MG63 showed the highest migration and invasion activity, we chose this cell line to establish a stable DRP5 knockdown cell line (shDRP5) to further analyze the role of DRP5 in osteosarcoma development. A DRP5 shRNA lentivirus was designed, and the scrambled sequence was used as the NC (shCtrl). After successful generation of the two stable cell lines, protein expression was assessed by western blotting. DRP5 expression was significantly downregulated in osteosarcoma shDRP5 cells, indicating the efficient knockdown effect (Fig. 2A and B). DRP5 silencing was associated with the downregulation of MMP‐2 and MMP‐9, two commonly used markers of migration and invasion (Fig. 2A and B). Next, cell migration was assessed by the wound healing assay and growth rate by the MTS assay. Wounds generated on shDRP5 MG63 osteosarcoma cells did not heal for 48 h, at which time wounds in the shCtrl cells were almost completely healed (Fig. 2C and D). In addition, DRP5 knockdown significantly suppressed the growth of MG63 cells (Fig. 2E). Migration and invasion assays showed that DRP5 knockdown significantly inhibited the migration and invasion of MG63 cells compared with those of normal and shCtrl control cells (Fig. 3A–D). The effect of DRP5 knockdown on cell cycle progression was assessed by flow cytometry. DRP5 knockdown induced cell cycle arrest at G0/G1 phase (Fig. 3E–F). Taken together, these data suggested that DRP5 is involved in osteosarcoma development and plays a critical role in the migration and invasion of osteosarcoma cells in vitro.

### DRP5 silencing suppressed the growth of osteosarcoma cells in vivo

**Figure 4:**
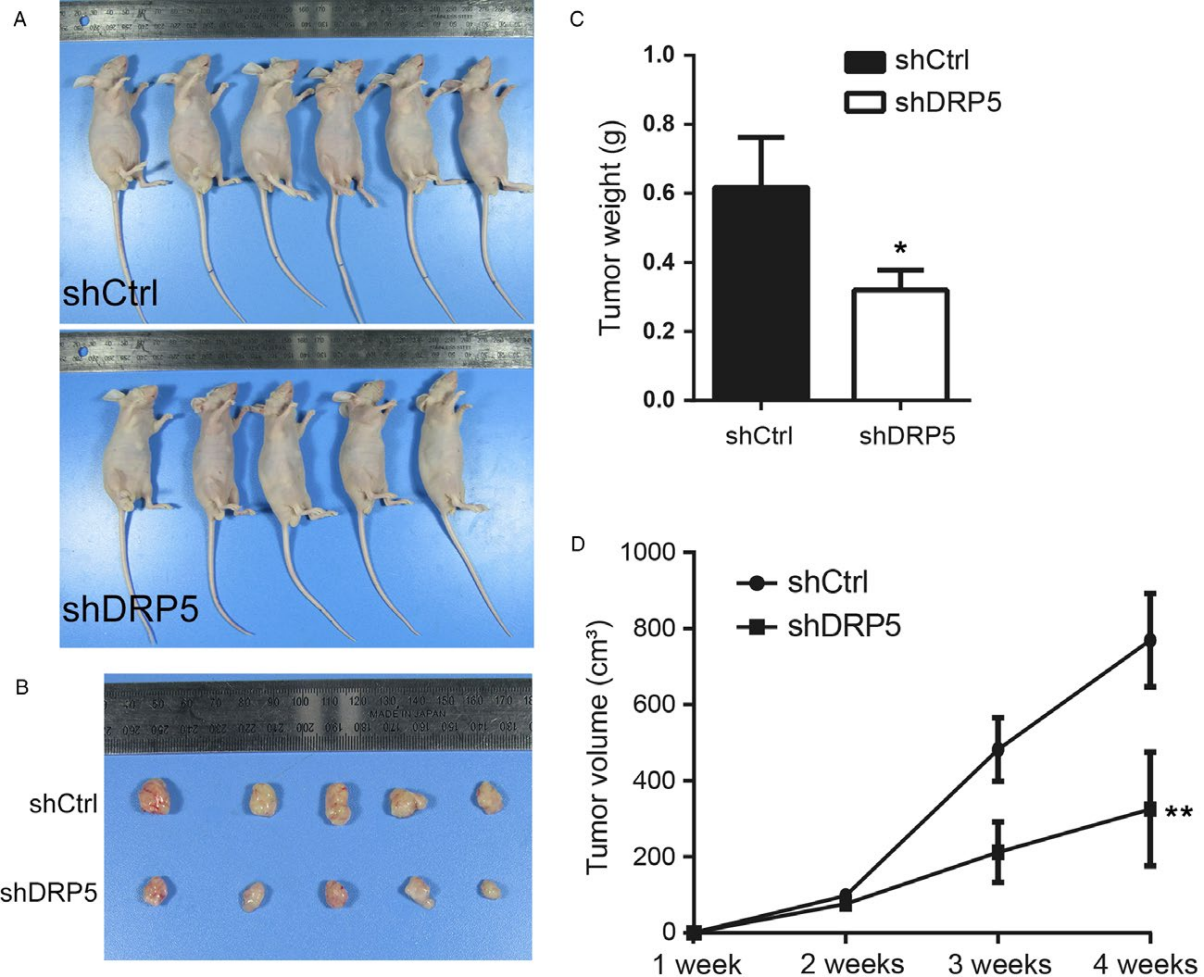
Inhibition of DRP5 suppresses tumor growth in vivo. The DRP5 silenced shDRP5 stable cell line or the control shCtrl cell line was implanted into nude mice for 4 weeks. Images of mice and isolated tumors are shown in (**A**) and (**B**). The tumor weight is shown in (**C**), and tumor volume was monitored every week and the results are shown in (**D**). * denotes P < 0.05, ** denotes P < 0.01 versus shCtrl group. A two‐sided ANOVA with a Bonferroni post hoc test was used for statistical analysis.

Next, we investigated whether DRP5 inhibition affected tumor cell growth in an osteosarcoma orthotropic animal model in vivo. shDRP5 MG63 and shCtrl MG63 cells were inoculated subcutaneously into the flanks of nude mice. Tumor‐bearing mice were sacrificed on day 30, and assessment of tumors showed that DRP5 knockdown significantly suppressed tumor growth compared with that in control mice (Fig. 3A and B). The tumor weight (Fig. 4C) and tumor volume (Fig. 4D) were markedly decreased in shDRP5 mice compared with those in the control group. Taken together, these data indicated that DRP5 is involved in osteosarcoma development, and inhibition of DRP5 can suppress tumor cell growth of osteosarcoma in vitro and in vivo.

### High DRP5 protein expression is associated with poor clinical outcomes in patients with osteosarcoma

**Figure 5:**
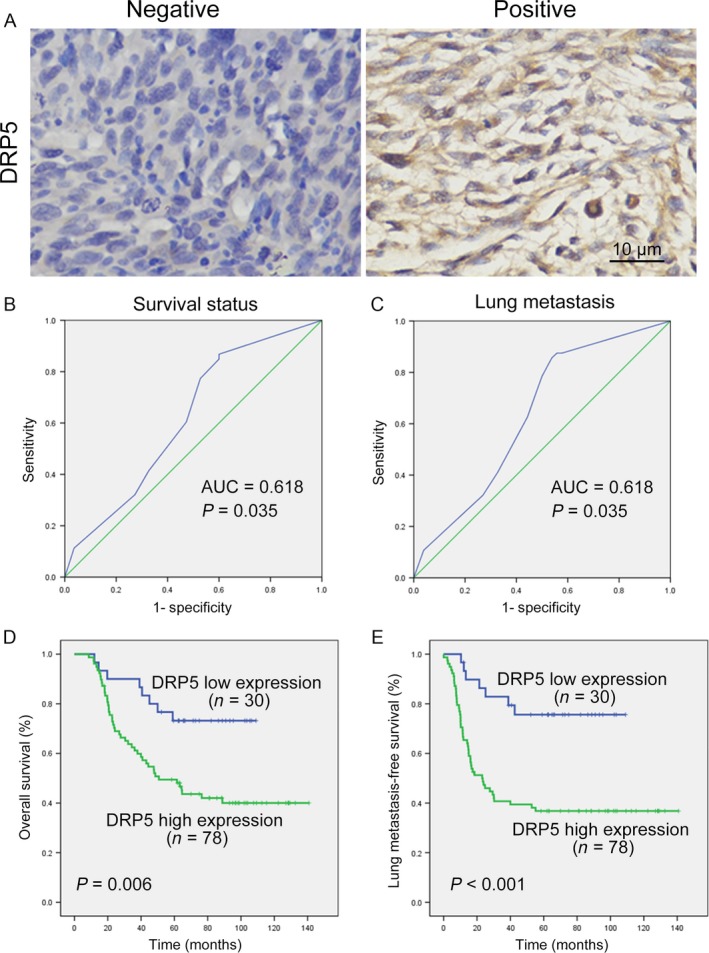
Upregulated DRP5 proteins in tumor tissues of osteosarcoma patients implies poor clinical prognosis. Immunohistochemical staining was used to detect DRP5 protein expression in osteosarcoma tissues (n = 108). Representative negative and positive images are shown (**A**). ROC curve analysis was used to determine the cutoff value for high expression of the DRP5 protein in lung osteosarcoma tissues. The sensitivity and specificity for overall survival (**B**) and lung metastasis (**C**) were plotted. Kaplan–Meier analysis of overall survival (**D**) and lung metastasis‐free survival (**E**) with high or low DRP5 expression.

Because our results suggested that DRP5 acts as an oncoprotein in osteosarcoma, the clinical significance of DRP5 was evaluated in primary human osteosarcoma tissue samples. Surgical specimens from 108 cases of osteosarcoma collected between January 2005 and December 2009 were immunohistochemically stained for DRP5 (Table 1). As shown in Figure 4A, the DRP5 protein localized to the nucleus and cytosol of cancer cells. The correlations between DRP5 expression and the clinical characteristics of patients are presented in Table 2. Because osteosarcoma has a high rate of lung metastasis 25, 26, we collected data on lung metastasis in osteosarcoma patients. The ROC curves for DRP5 showed the point on the curve closest to 0.0 and 1.0, which maximized both the sensitivity and specificity for overall survival (Fig. 5B) and lung metastasis (Fig. 5C). Osteosarcoma patients with high DRP5 protein expression had a significantly shorter overall survival time (Fig. 5D) and lung metastasis‐free survival time (Fig. 5E) than those with low DRP5 expression. These data suggest that high DRP5 protein expression is associated with poor clinical outcomes in patients with osteosarcoma.

**Table 1:** Clinical characteristics of 113 osteosarcoma patients

	Total = 108	Percentage (%)
Age (years)		
Average	19	
Range	6–51	
Gender		
Male	69	63.8
Female	39	36.2
Location		
Distal femur	59	54.6
Proximal tibia	25	23.1
Proximal humerus	12	11.1
Proximal femur	5	4.6
Other	7	6.5
Enneking score		
IIB	82	75.9
III	26	24.1
Relapse		
Yes	9	8.3
No	99	91.7
Lung metastasis		
Yes	58	53.7
No	50	46.3
Death		
Yes	55	50.9
No	53	49.1

**Table 2:** The association of DRP5 expression with patient clinicopathological characteristics in 108 osteosarcoma tissues

	Number	DRP5 expression level		*P* value^a^
		High	Low	
Age				0.865
≤20	78	33	45	
21–40	27	14	13	
>40	3	2	1	
Gender				0.245
Male	69	33	37	
Female	39	24	15	
Location				0.634
Distal femur	59	36	23	
Proximal tibia	25	12	13	
Proximal humerus	12	5	7	
Proximal femur	5	2	3	
Other	7	4	3	
Enneking score				0.741
IIB	82	38	44	
III	26	15	11	
Relapse				0.635
Yes	9	5	4	
No	99	51	48	
Lung metastasis				0.021
Yes	58	33	25	
No	50	29	31	
Death metastasis				0.006
Yes	55	29	26	
No	53	23	30	

a Chi‐square test.

## Discussion

Osteosarcoma is the most common primary malignant tumor of the bone and is associated with a high rate of pulmonary metastasis, which is the most critical prognostic factor for survival. Approximately, 15–25% of osteosarcoma patients develop metastatic disease, leading to failure of treatment. The 5‐year survival rate of patients with metastasis from osteosarcoma is approximately 10–20%, whereas the survival rate is 60–70% in patients without metastasis 27, 28. However, the underlying molecular mechanisms remain largely unknown. The identification of novel proteins involved in osteosarcoma development has potential applications in anticancer treatment. To elucidate the mechanism underlying the progression of human osteosarcoma, we collected specimens from osteosarcoma patients and used osteosarcoma cell lines to evaluate the role of DRP5 for the first time. Our results showed that DRP5 was significantly overexpressed at the mRNA and protein levels in osteosarcoma cell lines, and this upregulation was related to the migration and invasion activities of osteosarcoma cells. Knockdown of DRP5 markedly suppressed the expression of MMPs and inhibited the migration and invasion of osteosarcoma cells. Moreover, DRP5 silencing inhibited tumor growth in nude mice in vivo. DRP5 protein expression was high in patients with osteosarcoma and associated with significantly shorter overall survival and shorter lung metastasis‐free survival rates than those of patients with low DRP5 levels. These data suggested the important role of DRP5 in osteosarcoma development and its potential role in osteosarcoma metastasis.

CRMPs are highly expressed in the developing and adult nervous system 6, 7, 8 and function in the regulation of neurite outgrowth and development, axonal guidance, and neuronal polarity and development. All CRMP proteins (CRMP 1–5) associate with the cytoskeleton 15, 29 to promote the migration of filopodia and lamellipodia, which is important for cancer metastasis and invasion 30. In recent years, CRMP proteins have been implicated in the pathologies of a variety of human cancers. CRMP‐1 is suggested to be a cancer suppressor 17, 31, 32, while LCRMP‐1 functions to promote cancer metastasis 19, 33, 34. CRMP‐2 was suggested as a prognostic marker and candidate therapeutic target in NSCLC and colorectal carcinoma 20, 35, 36, 37. Regarding DRP5, its neuronal autoantibody was reported to be related with patients at risk for lung carcinoma 22, 38, 39. The function of other CRMP proteins in cancer development remains to be elucidated. Although evidence of the role of CRMP proteins in cancer is accumulating, the underlying mechanisms need to be further explored.

MMPs are involved in epithelial–mesenchymal transition and in extracellular matrix degradation 40. In the current study, knockdown of DRP5 resulted in a remarkable downregulation of MMP‐2 and MMP‐9, which was consistent with the reduced numbers of osteosarcoma cells with migratory and invasive activities. These results suggested that DRP5 functions upstream of MMPs to regulate the migration and invasion of osteosarcoma cells. The interaction of DRP5 with the cytoskeleton suggests that investigation of the relationship between DRP5 and tubulin and actin during osteosarcoma development is warranted. CRMP proteins are regulated by a variety of posttranscriptional modifications. They can be phosphorylated by many kinases, most notably GSK‐3*β* 41, cyclin‐dependent kinase 5 (Cdk5) 42, and Rho‐associated kinase 43. All members of the CRMP family, including DRP5, contain a Cdk5 phosphorylation consensus site. Thr514 phosphorylated CRMP‐2 in samples obtained from patients with localized NSCLC was shown to regulate the mitosis of cancer cells, and CRMP‐2 phosphorylation was suggested as a prognostic marker 20. The functions of the upstream kinases of DRP5 and the phosphorylation status during osteosarcoma progression remain to be explored, which is worth investigating in future studies.

In conclusion, the present study describes the relevance of DRP5 during osteosarcoma development. DRP5 was upregulated in osteosarcoma specimens and cell lines and shown to function via the downstream MMPs. Inhibition of DRP5 suppressed the growth of cancer cells in vitro and in vivo, and high expression levels of DRP5 were associated with poor prognosis in osteosarcoma patients. These data suggested that DRP5 is a prognostic marker and potential new target for cancer therapy.

## Conflict of Interest

The authors declare no conflicts of interest.
